# A scoping review of the wide array of Alzheimer’s disease theories and hypotheses supports a multifactorial etiology

**DOI:** 10.1002/alz.088873

**Published:** 2025-01-03

**Authors:** Simon Duchesne, Louis‐Simon Rousseau, Olivier Potvin, Florence Belzile, Laurie‐Ann Welch, Béatrice Cournoyer, Marianne Arseneau, Véronick Lapierre, Sara‐Maude Poulin, Carol Hudon

**Affiliations:** ^1^ Centre de recherche de l’Institut Universitaire de Cardiologie et de Pneumologie de Québec, Quebec, QC Canada; ^2^ Radiology and Nuclear Medicine Department, University Laval, Quebec City, QC Canada; ^3^ CERVO Brain Research Centre, Quebec City, QC Canada; ^4^ Université Laval, Quebec City, QC Canada; ^5^ Université Laval, Quebec, QC Canada; ^6^ CERVO Research Centre, Québec, QC Canada

## Abstract

**Background:**

There is a common agreement that Alzheimer’s disease (AD) is inherently complex; otherwise, a general disagreement remains on its etiological underpinning, with numerous alternative hypotheses having been proposed. Our objective was to perform a scoping review of original manuscripts describing hypotheses and theories of AD published in past decades.

**Methods:**

We reviewed 127 original manuscripts that fulfilled our inclusion criteria out of more than 13,807 references extracted from open databases (from inception to 14 Sept 2023). Each entry was characterized as having a single or multifactorial focus and assigned to one of 15 theoretical groupings. Impact was tracked using open citation tools.

**Results:**

We found that three quarter of studies proposed a hypothesis characterized as being single‐focus (97/127), with the most important theoretical groupings being the Amyloid group, followed by Metabolism and Mitochondrial dysfunction, then Infection and Cerebrovascular. Three stages can be discerned in terms of hypotheses generation. The first phase (∼1980‐1995) included the establishment of the main thrusts that have endured to this day (Amyloid, Glial, Infection, Inflammation, Metabolism, Oxidative stress, and Proteinopathies hypotheses; multifactoriality; and neurotoxicity). In the second phase (1995‐2005), the importance of the Cerebrovasculature, Mitochondrial dysfunction, and Neurotransmitters were recognized. Lately (2005‐2023), evidence towards Genetics (outside of the autosomal dominant form of AD), and especially Gut/Brain interactions came to the fore. Impact, as measured by total citations count, followed a similar pattern. Out of a total of 19,996 citations for the 127 papers, the most cited single‐focused grouping was Amyloid (52%), followed by Mitochondrial dysfunction (6%, reaching 10.0% when adding Metabolism articles), then Cerebrovascular (5%) and Oxidative Stress (4%).

**Conclusion:**

When viewed together, these multi‐faceted reports reinforce the notion that AD affects multiple sub‐cellular, cellular, anatomical, and physiological systems at the same time but at varying degree between individuals. Future work should integrate these competing views into a comprehensive, systemic framework able to capture individual and combinatorial factor evolutions and interactions, to manage its inherent complexity.

